# Genomic Analysis of Selected Maize Landraces from Sahel and Coastal West Africa Reveals Their Variability and Potential for Genetic Enhancement

**DOI:** 10.3390/genes11091054

**Published:** 2020-09-07

**Authors:** Charles Nelimor, Baffour Badu-Apraku, Ana Luísa Garcia-Oliveira, Antonia Tetteh, Agre Paterne, Assanvo Simon-Pierre N’guetta, Melaku Gedil

**Affiliations:** 1 West African Science Service Centre on Climate Change and Adapted Land-use (WASCAL), Université Felix Houphouët Boigny, Abidjan 22 BP 461, Côte d’Ivoire; nelimor.c@edu.wascal.org; 2 International Institute of Tropical Agriculture, Ibadan 200001, Nigeria; P.Agre@cgiar.org (A.P.); m.gedil@cgiar.org (M.G.); 3 Department of Bioscience, Université Felix Houphouët Boigny, Abidjan 22 BP 461, Côte d’Ivoire; nguettaewatty@gmail.com; 4 Excellence in Breeding (EiB) Platform, International Maize and Wheat Improvement Center (CIMMYT), ICRAF House, Nairobi 00100, Kenya; a.oliveira@cgiar.org; 5 Department of Biochemistry and Biotechnology, Kwame Nkrumah University of Science and Technology, University Post Office Box PMP, Kumasi 00233, Ghana; aytetteh@gmail.com

**Keywords:** landraces, genetic diversity, population structure, West Africa, maize improvement, DArTseq markers

## Abstract

Genetic adaptation of maize to the increasingly unpredictable climatic conditions is an essential prerequisite for achievement of food security and sustainable development goals in sub-Saharan Africa. The landraces of maize; which have not served as sources of improved germplasm; are invaluable sources of novel genetic variability crucial for achieving this objective. The overall goal of this study was to assess the genetic diversity and population structure of a maize panel of 208 accessions; comprising landrace gene pools from Burkina Faso (58), Ghana (43), and Togo (89), together with reference populations (18) from the maize improvement program of the International Institute of Tropical Agriculture (IITA). Genotyping the maize panel with 5974 DArTseq-SNP markers revealed immense genetic diversity indicated by average expected heterozygosity (0.36), observed heterozygosity (0.5), and polymorphic information content (0.29). Model-based population structure; neighbor-joining tree; discriminant analysis of principal component; and principal coordinate analyses all separated the maize panel into three major sub-populations; each capable of providing a wide range of allelic variation. Analysis of molecular variance (AMOVA) showed that 86% of the variation was within individuals; while 14% was attributable to differences among gene pools. The Burkinabe gene pool was strongly differentiated from all the others (genetic differentiation values >0.20), with no gene flow (Nm) to the reference populations (Nm = 0.98). Thus; this gene pool could be a target for novel genetic variation for maize improvement. The results of the present study confirmed the potential of this maize panel as an invaluable genetic resource for future design of association mapping studies to speed-up the introgression of this novel variation into the existing breeding pipelines.

## 1. Introduction

Maize (*Zea mays* L.) is one of the most important cereal crops consumed in sub-Saharan Africa (SSA) and an essential component of livestock feed in the developed as well as developing world. Approximately 10,000 years ago in Central Mexico, maize was domesticated from its ancestor, the wild grass-teosinte (*Zea mays* spp. *parviglumis*) [1]. Post-domestication of teosinte led to the transfer of beneficial adaptive genomic regions into common maize [2]. Mutations together with recombination events, either due to natural or farmer-mediated selection, then generated novel allele combinations [3,4]. Thus arose the “landraces”, traditional varieties selected by farmers for adaptation to local conditions and food preferences [5]. The landraces, though typically low in yield, are invaluable sources of diversity that could be drawn upon to broaden the genetic base of elite maize germplasm, and to further enhance adaptation to changing environments and pathogens [6]. Indeed, earlier breeders identified and composited the most productive landraces into genetically diverse populations, forming the foundation of inbred line development and pedigree breeding [7]. Modern breeding practices, in which a narrow range of inbred lines are included in crossing programs, have narrowed the genetic base of most cultivated crops, which have negatively affected the adaptability of the crop to changing climates, devastating pathogens, and insect-pests [8]. In order to provide a buffer against the possible effects of novel threats, it is essential to broaden the genetic base of breeding populations by introgressing an enlarged pool of beneficial alleles.

The high selection pressure under low input and climatically stressed maize growing environments in Africa is likely to have resulted in local adaptations with potential value for breeding stress tolerant and nutritionally enriched varieties [9]. Indeed, there are examples of successful use of local African genetic resources of maize in the development of varieties such as Katumani in Kenya and Longe-5 from Uganda [9]. However, while the New World maize gene pool is well represented in genebanks [10] and well characterized [3], there are collections of African maize germplasm without adequate data of their genetic make-up [9]. This missing genetic data makes searching for promising landraces within the African maize genebank collections like “finding the proverbial needle in a haystack” [11]. Therefore, to develop improved climate resilient maize varieties for SSA, efficient characterization, identification, and utilization of climatically adapted local African maize germplasm is a crucial prerequisite [9].

Recently, we phenotypically characterized a panel of maize landraces originating from Burkina Faso, Ghana, and Togo, which cover a wide range of climatic conditions classified as Sahel and coastal West Africa (WA) [12]. The study revealed that the maize panel varied considerably in flowering date, plant architecture, yield and yield related traits, and other characteristics. These differences allowed the formation of five distinctive morphological groups [12]. The Sahel gene pool was highly distinct and was considered a valuable resource for future genetic enhancement. However, phenotypic variation can be confounded by the environment and a high degree of plasticity [13]. Given that gene flow from multiple introductions may have shaped the population structure of African maize, diversity analysis of this panel using state-of-the-art genotyping techniques could help provide deep insights into the complexity of its genetic architecture and composition.

The assessment of genetic diversity by genotyping-by-sequencing (GBS) provides robust estimates of diversity and has been increasingly adopted as a fast, high-throughput, and affordable tool for whole-genome genetic diversity analysis in large germplasm sets [14]. The diversity array technology sequence (DArTseq) markers, characterized by high marker coverage, call rates, and scoring reproducibility, has emerged as a useful GBS approach for assessing genetic diversity and population structure in various crops including wheat [15,16], rice [17], watermelon [18], common bean [19], and maize [20,21]. The objective of this study was to examine the genetic diversity and population structure of a maize panel comprising landraces from Sahel and coastal Africa together with a reference population using the GBS-DArTseq approach. The relevance of our results for further exploration and utilization of local genetic resources of maize in Africa is discussed in this manuscript.

## 2. Materials and Methods

### 2.1. Plant Material 

We analyzed 208 maize accessions obtained from international and national gene banks in Africa (Supplementary Table S1). The maize panel comprised 190 landraces representing gene pools from Burkina Faso (58), Ghana (43), and Togo (89) (Supplementary Figure S1). The landraces from Burkina Faso and Togo were sourced from the gene bank at the International Institute of Tropical Agriculture (IITA), Ibadan, Nigeria, whereas those from Ghana were provided by the Plant Genetics Resources Institute (PGRI) at Bunso, Ghana. The majority of these landraces were collected from farmers’ fields in the 1970s and 1980s. However, the eco-geographical data of the collection sites of the landraces were not available. The study also included a diverse set (18) of drought- and heat-tolerant open pollinated populations (hereafter referred to as reference populations) developed by the Maize Improvement Program at IITA (MIP-IITA), Ibadan, Nigeria.

### 2.2. DNA Isolation and Genotyping Analysis

For each accession, total genomic DNA was isolated from bulked leaf composites from 15 seedlings at two weeks old according to the DArT protocol (https://www.diversityarrays.com/orderinstructions/plant-dna-extraction-protocol-for-dart/). The quality of each DNA sample was visualized by electrophoresis on 0.8% agarose gel, and the purified DNA was further quantified using a nano-drop spectrophotometer (Thermo Scientific, Wilmington DE, USA). Certified DNA samples were then sent to the Integrated Genomic Service and Support (IGSS) genotyping platform, Nairobi, Kenya, for genotyping. High-throughput genotyping was conducted in 96 plex DArTseq protocol, and SNPs were called using the DArT’s proprietary software, DArTSoft, as previously described [22]. Reads and tags found in each sequencing result were aligned to the *Zea mays* L. genome reference, version *AGPV3* (B73 Ref-Gen v4 assembly) [23].

### 2.3. Data Analysis

A total of 47,441 putative DArTseq markers were generated from the 208 maize panel. Prior to further analysis, the raw data set was filtered to remove markers with call rate <0.8, minor allele frequency (MAF) <0.05, and unmapped SNP markers. Thereafter, markers with no missing rate were retained using the TASSEL software version 5.2.12 [24]. The retained markers were subjected to various genetic diversity analyses including basic diversity statistics such as polymorphic information content (PIC), MAF, observed heterozygosity (H_o_), and expected heterozygosity (H_e_) using PowerMarker v. 3.2.5 [25]. The population structure of the maize panel was inferred using the Admixture model-based clustering algorithm implemented STRUCTURE 2.3.4 [26]. The *adhoc* number of clusters (k) was varied from 1 to 12, with 10,000 burn-in steps, followed by 10,000 Markov chain Monte Carlo simulations, as previously described [20,27]. For each k, ten independent iterations were implemented. The most likely number of k was determined by the ad hoc Δ k statistics [28] embedded in Structure Harvester [29]. Accessions with membership proportions (Q-value) ≥80% were assigned to groups, while those with membership probabilities less than 80% were designated as admixtures [30]. The population structure of each gene pool (Burkinabe, Ghanaian, Togolese, and reference populations) were also estimated as described above. A discriminant analysis of principal components (DAPC) was carried out on the 208 maize panel using the first 40 principal components using the adegenet R package [31]. Membership probabilities of the individuals for the different gene pools were estimated using the “find cluster” function implemented in adegenet. Further, principal coordinate analysis (PCoA) was conducted to reveal the genetic relationships among the maize accessions using GenAlEx v. 6.503 software [32]. An unrooted neighbor-joining (NJ) tree was constructed by following the procedure of Nei [33] with 1000 bootstrap replicates in PowerMarker v3.25 [25]. The resulting NJ tree was visualized in Molecular Evolutionary Genetics Analysis (MEGA) software version X [34] and edited using Figtree software v.1.4.4 [35]. Genetic relationships within each maize gene pool were elucidated through construction of an unrooted NJ tree, as described above. Analysis of molecular variance (AMOVA) was estimated in GenAlEx v. 6.503 [32] to partition components of genetic variance among and within the populations (k). Calculation of pairwise genetic differentiation statistics (*F_ST_*) and haploid number of migrants (Nm) between gene pools was performed using GenAlEx v6.503 [32] with 999 permutations. *F_ST_* measures the amount of genetic variance that can be explained by population structure based on Wright’s F-statistics [36], while *Nm* $\;=\left[\left(1/F_{ST}\right)-1\right]/4$. An *Nm* value less than 1 indicates limited gene exchange among subpopulations [36].

## 3. Results

### 3.1. Analysis of Genetic Diversity Parameters

Out of the total 47,441 putative DArTseq markers, 5974 were retained after filtering. The GBS-DArTseq markers were unequally distributed across the ten chromosomes of the 208 maize panel. Chromosome 1 had the highest number of markers (905), while chromosome 10 had the least (422) (Supplementary Figure S2). H_e_, H_o_, MAF, and PIC values estimated for the entire maize panel (208 accessions) averaged 0.36, 0.50, 0.28, and 0.29, respectively (Table 1). The average values of H_e_, Ho, MAF, and PIC were 0.30, 0.41, 0.23, and 0.24, respectively, for the Burkinabe gene pool, and 0.32, 0.34, 0.23, and 0.26, respectively, for the Ghanaian gene pool. The average H_e_, H_o_, MAF, and PIC values of the Togolese gene pool were 0.36, 0.50, 0.27, and 0.28, respectively. Furthermore, the He, H_o_, MAF, and PIC values of the reference population averaged 0.36, 0.47, 0.28, and 0.29, respectively. For the landraces as a group, H_e_, H_o_, MAF, and PIC values averaged 0.37, 0.50, 0.28, and 0.29, respectively.

### 3.2. Population Structure and Genetic Relationships

**Figure 1:**
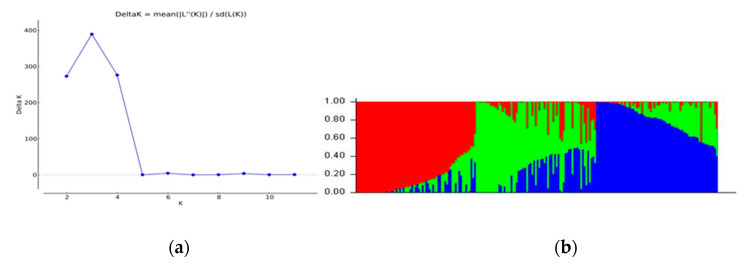
Graphical representation of the population structure of the 208 maize panel. (**a**) Plot of mean likelihood of delta K against the number of K groups. The highest peak observed at K = 3 signifies the grouping of accessions into three groups, while the small peak at K = 2 and 4 signifies further grouping of accessions into two and four groups, respectively. (**b**) Subpopulations at K = 3. The colors represent three subpopulations of 208 accessions. The separation of accessions into subpopulation 1 (red), 2 (green), and 3 (blue) was based on membership coefficient ≥80%.

**Figure 2:**
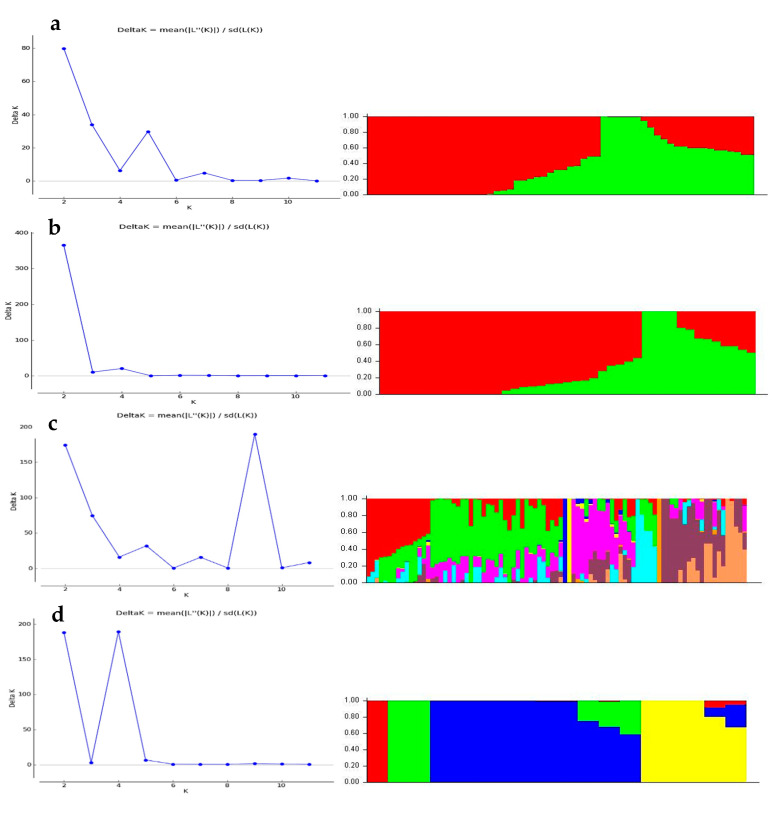
Population structure of Burkinabe (**a**), Ghanaian (**b**), and Togolese (**c**) landrace gene pools, including a reference population (**d**) at K = 2, K = 9, and K = 4, respectively.

The model-based simulation of population structure analysis of the maize panel (208 accessions) showed that the delta K values from the mean log-likelihood probabilities plateaued at K = 3 (389.43), followed by K = 4 (276.33), and K = 2 (273.07) (Figure 1a). At K = 3, the 208 maize panel was divided into three sub-populations (Figure 1b). Using an 80% membership probability threshold, 122 accessions (58.65%) were successfully assigned to the three subpopulations. In comparison, 86 accessions with a probability of associations less than 80% were designated as an admixed population (Supplementary Table S2). Subpopulation 1 was the most uniform (membership coefficient averaged, 90%), and it contained 53 landraces (49 from Burkina Faso, 3 from Togo, and 1 from Ghana). Subpopulations 2 and 3, which constituted 12.5% and 20.67% of the panel, respectively, were admixtures of Ghanaian and Togolese landraces, together with reference populations. Specifically, subpopulation 2 consisted of 26 accessions, 10 reference populations, and 11 and 5 Togolese and Ghanaian landraces, respectively. Subpopulation 3 comprised 24 and 15 landrace accessions from Ghana, and Togo, respectively, and 4 accessions from the reference population. The admixed group contained 60, 13, and 9 landraces from Togo, Ghana, and Burkina Faso, respectively, plus 4 reference populations (Supplementary Table S2). The additional smaller peaks observed at K = 4 (276.33) and K = 2 (273.07) implied the presence of subgroups within the three major groups (Figure 1). Therefore, an independent STRUCTURE run was performed for each gene pool. Sub-clustering of the Burkinabe and Ghanaian gene pools both yielded a sharp peak at K = 2 (Figure 2a,b). Sub-clustering the reference populations and Togolese landraces showed the highest peak at K = 3, and K = 9, respectively (Figure 2c,d). A substantial degree of admixture was observed for each gene pool (Supplementary Table S3).

**Figure 3:**
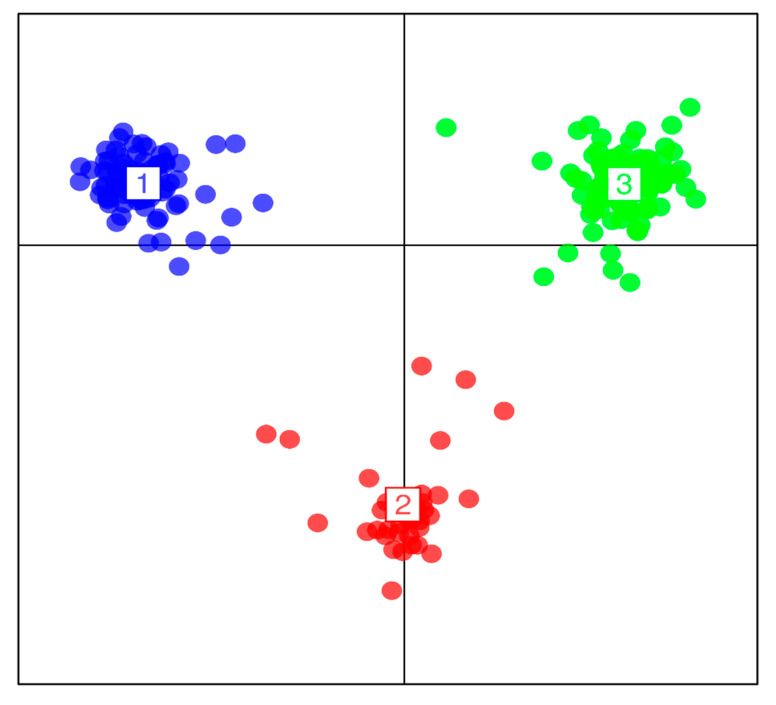
Discriminant analysis of principal components (DAPC) using 5974 DArTseq markers. The axes represent the first two linear discriminants (LD). Each color represents a cluster, while each dot represents an individual. Numbers represent the different subpopulations identified by DAPC analysis.

Using the Bayesian information criterion (BIC) implemented in DAPC, a maximum of K = 3 was obtained, which corresponded to three groups of maize accessions in the panel (Figure 3). Estimation of the cluster membership revealed that cluster three had the highest number of accessions (94) followed by cluster two with 77 accessions, and cluster one with the smallest number of accessions (37). Of the 94 accessions in cluster three, 58 (61.70%) and 29 (30.85%) were landraces from Togo and Ghana, respectively, including six reference populations and the landraces from Burkina Faso (Supplementary Table S4). All the accessions in cluster two were landraces from Burkina Faso (57), Togo (17), and Ghana (3). Of the 37 accessions in cluster 1, 14 (37.84%) were Togolese landraces, 12 (32.43%) were from the reference populations, while 11 (29.73%) were Ghanaian landraces.

**Figure 4:**
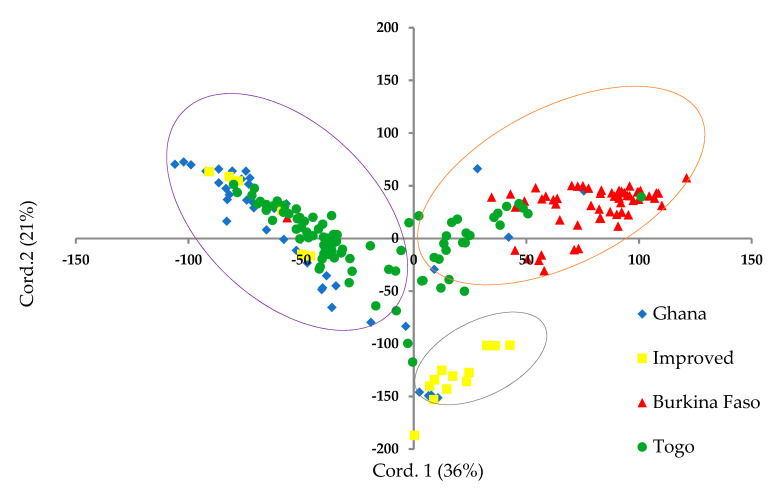
Cluster analysis of 208 maize accessions using principal coordinate analysis (PCoA). Accessions are colored according to origin. The orange, ash, and violet circles represent subpopulation 1, 2, and 3, respectively.

Further investigation of the genomic structure of the maize panel using the PCoA indicated three subpopulations as per the STRUCTURE simulation and DAPC analyses (Figure 4). The total amount of genetic variation explained by the first two principal coordinates was 57%. The PCoA clearly separated subpopulation 3 (by PCo2), which showed a higher degree of admixture between Ghanaian and Togolese landraces, including six reference populations and a landrace from Burkina Faso. The other two subpopulations appeared to be distributed along PCo1. Although some degree of overlap among landrace gene pools was shown in subpopulation 1, located at the upper extreme of PCo1, ~75% were Burkinabe landraces. Subpopulation 2 distributed along the lower extreme of PCo1 was the most distant of the three, comprising the majority of the reference populations and four Ghanaian landraces.

**Figure 5:**
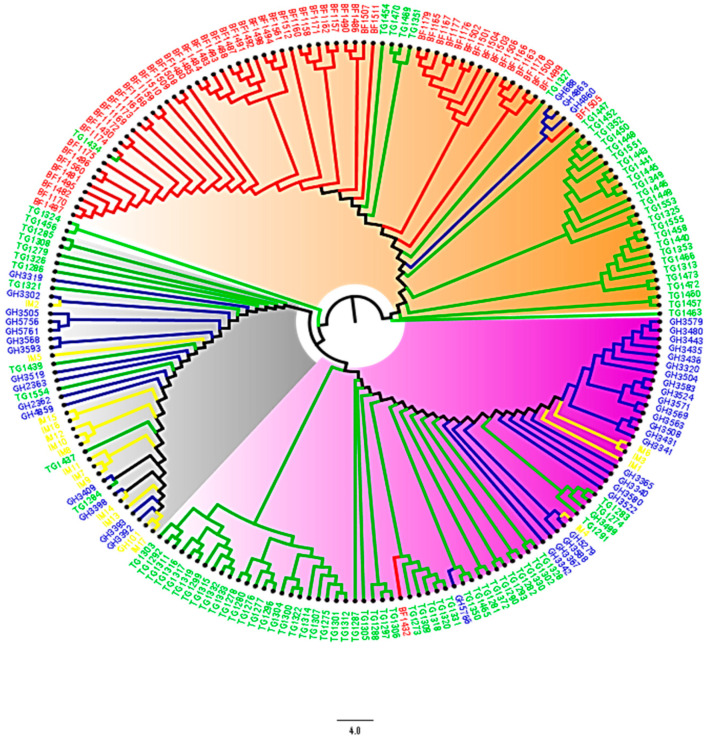
Phylogenetic tree estimated through the neighbor-joining method for 208 maize accessions from West Africa. The green, red, blue, and yellow clades and taxa represent Togolese, Burkinabe, Ghanaian, and reference populations, respectively. The orange, violet, and ash highlights represent subpopulations 1, 2, and 3, respectively.

**Figure 6:**
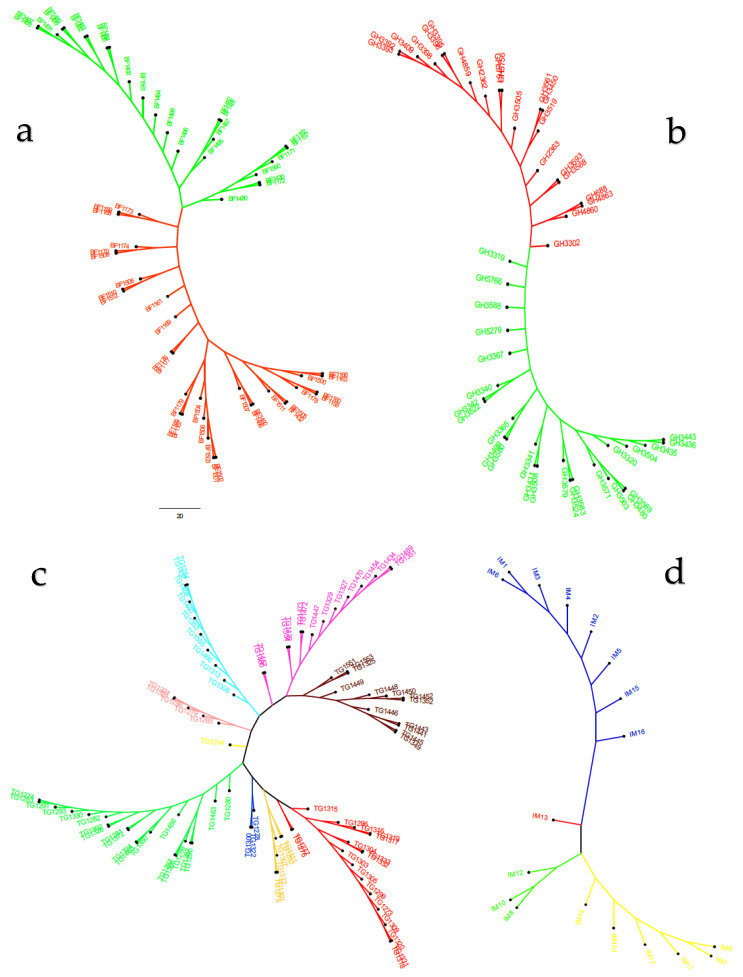
Unrooted neighbor joining tree depicting genetic relationships among Burkinabe (**a**), Ghanaian (**b**), and Togolese (**c**) landrace gene pools, and a reference population (**d**). Colors of each tree represent genetic groups.

As per the STRUCTURE, DAPC, and PCoA results, the NJ phylogenetic tree also showed three sub-populations with higher degrees of admixture among Ghanaian and Togolese landraces, and reference populations (Figure 5). The neighbor-joining tree performed for each gene pool divided the Burkinabe and Ghanaian gene pools into two main clusters (Figure 6b). The Togolese landraces and the reference populations were grouped into nine and three clusters, respectively (Figure 6b,c).

### 3.3. Analyses of Molecular Variance, Genetic Differentiation, and Gene Flow among Gene Pools

The AMOVA revealed that 14% of the total variation was found among gene pools, while the rest (86%) was within gene pools (Table 2). The overall *F_ST_* value of the maize panel was 0.21, and the Nm value was 1.58. As shown in Table 3, the Burkinabe gene pool had the highest *F_ST_* value (0.28), and the Ghanaian and Togolese gene pools had the lowest (0.18, each). The pairwise *F_ST_* values ranged from 0.14 (Ghanaian vs. Togolese) to 0.31 (Burkinabe vs. reference populations). Similarly, Nm values between gene pools varied from 0.98 (Burkinabe vs. reference populations) to 2.83 (Ghanaian vs. reference populations). The Nm value between the Ghanaian and Togolese gene pools was 2.63.

## 4. Discussion

A well-characterized and diverse germplasm is an essential requisite for genetic enhancement of crops. In this study, we applied GBS technology to explore the genetic diversity and population structure of a maize panel comprising landrace gene pools from Burkina Faso, Ghana, and Togo, plus a reference population from IITA-MIP. The results of the estimated diversity indices revealed ample genetic diversity within the maize panel indicated by average H_e_ (0.36) and H_o_ (0.5). The He obtained in this study was comparable to the 0.36 reported for provitamin A (PVA) quality protein maize (QPM) germplasm from IITA-MIP [21] but was higher than that reported for maize landraces from Eastern Africa (H_e_ = 0.25), Western Africa (H_e_ = 0.18), and Sahel Africa (H_e_ = 0.24) [9] as well as tropical maize breeding populations (H_e_ = 0.22) [27] including IITA early-maturing white inbred lines [20]. Characterization of the Burkinabe, Ghanaian, and Togolese maize pools showed different values for the estimated diversity indices. The results indicated that the Togolese gene pool (H_e_ = 0.36, H_o_ = 0.50) contained slightly higher diversity than the Burkinabe (H_e_ = 0.30, Ho = 0.41) and Ghanaian (H_e_= 0.32, H_o_ = 0.34) landrace pools. Further, the low variation in the genetic indices identified between the landraces as a group, and the reference populations showed that the two germplasm sets possessed similar genetic diversity (Table 1). These results agreed with previous findings that tropical maize germplasm is highly diverse with H_e_ > 0.3 [37,38,39]. The mean PIC obtained in the present study, 0.29 using 5974 DArTseq SNPs for the 208 maize accessions was higher than the 0.19 and 0.26 reported for tropical early-maturing maize inbred lines using 15,047 [30] and 7224 SNPs for a sample size of 94 and 134, respectively [20,27]. The discrepancies between the results of our study and those of earlier researchers may be due to the use of different genetic materials, the sample sizes, and the number of SNPs used. Nonetheless, the mean PIC value in this study was like the 0.29 recently reported for tropical PVA-QPM maize germplasm using 8171 DArTseq SNP markers [21].

The Evanno criterion employed for the model-based simulation of population structure identified the peak level of ΔK at K = 3 (Figure 1a), which depicted the presence of three genetically distinct subpopulations (Figure 1b). The proportion of admixed accessions (47%) in the maize panel, based on a membership probability threshold of 80%, suggested moderate genetic differentiation and gene flow. The DAPC, PCoA, and NJ phylogenetic analyses results all illustrated the existence of three subpopulations in the whole set of 208 maize accessions. Comparison of the results of the four complementary clustering methods (STRUCTURE, DAPC, NJ tree, and PCoA) revealed high consistency in the individuals assigned to each group, which reinforced the findings that the identified groups were indeed genetically distinct. The close proximity between Togolese and Ghanaian gene pools suggested high genetic relatedness of the two gene pools. This result was expected due to the geographical proximity of the two countries and the similarity of the climatic conditions. The Burkinabe gene pool largely diverged from all others, suggesting its adaptation to Sahel conditions, which is in agreement with its pattern of phenotypic diversity [12]. Multivariate analyses revealed high affinity of Ghanaian and Togolese landraces with the reference populations (Figure 1, Figure 3, Figure 4, Figure 5). It is likely that some of these accessions are not true landraces but, rather, old improved cultivars that were either recollected or wrongly classified, as farmers usually consider improved varieties cultivated over longer periods in a given area as landraces [40]. The grouping together of some landraces with the reference populations also suggested a pedigree relationship. Hence, it is possible that some of the landraces analyzed in this study were local varieties that were selected by earlier maize breeders in IITA, based on high grain yield, earliness, and resistance to the maize streak virus (MSV), and adaptation to the drought and heat stress as starting materials for the development of inbred lines that were later involved in cross-breeding (see http://r4dreview.iita.org/index.php/tag/maize-improvement/). The additional smaller peaks observed at K = 4 (276.33) and K = 2 (273.07) implied the presence of subgroups within the three major groups (Figure 1). Therefore, an independent STRUCTURE run was performed for each gene pool (Figure 2). The high degree of genetic admixtures within each landrace gene pool observed with ancestry share of <80% probably reflects considerable levels of gene flow or germplasm exchange. Results of previous studies have shown that such an admixture is not unusual in landraces from restricted geographical backgrounds [40].

According to Frankham et al. [41], an *F_ST_* value greater than 0.15 can be considered as significantly differentiating populations. Thus, in the present study, the overall *F_ST_* value (0.21) supported the presence of significant genetic divergence within the maize panel. Wright [36] reported that an Nm value less than 1 indicated limited gene exchange among populations. In the present study, the overall Nm value of 1.58 (Table 2) indicated that moderate genetic exchange or gene flow may have occurred, leading to the moderate genetic differentiation between gene pools. This observation was consistent with the AMOVA results (Table 2), which indicated that 14% of the total variation was accounted for by gene pool variations. This result is consistent with the findings of previous studies [42]. According to the *F_ST_* values, the Burkinabe gene pool was the most differentiated (Table 3), in agreement with its divergence as revealed by the clustering methods (STRUCTURE, PCoA, DAPC, and NJ analyses). The divergence between the reference populations and landraces varied among the different gene pools. In particular, the low affinity of the Burkinabe gene pool with the reference population (*F_ST_* = 0.31, Nm = 0.98) suggested little involvement of the original Sahelian gene pool in the development of the modern maize varieties presently grown in the sub region. This observation is biologically and historically meaningful since in West Africa, the reference maize gene pool called Composite Y [43], which was developed through recombination of 145 flint landraces of West Africa savannah zone, contained only 2% each of the genetic materials from Burkina Faso and Niger, as well as 1% of those from Senegal [44]. In the analyses of the isozyme variability in West African maize cultivars, Sanou et al. [45] showed that Burkinabe landraces were distinct, even though some levels of gene flow between them and an elite open pollinated variety (SR 22) developed by IITA in 1984 from CIMMYT Pop 22 and widely adopted in Burkina Faso [46] was observed. Therefore, the Burkinabe gene pool, having been grown and selected by farmers over many generations under warmer and drier conditions, could harbor novel and favorable alleles for improving maize for tolerance to drought and heat stresses. It is notable that in our earlier work on this maize panel, the high degree of tolerance of the Burkinabe landraces to drought, heat, and the combined heat and drought stresses was unrivalled [47,48]. The high genetic similarity observed between the Ghanaian and Togolese landraces was supported by their low *F_ST_* (0.14) and high Nm (2.63) values. This result further reflected the gene flow via seed exchanges and local preferences towards a given agrotype owing to similar climatic conditions. These may have significantly shaped the distribution of the genetic diversity within Ghanaian and Togolese maize landraces, as was previously suggested [12]. The *F_ST_* and Nm values (Table 3) suggested that the Ghanaian gene pool was closer to the reference populations, in agreement with PCoA stratification (Figure 4). Indeed, the reference set analyzed in this study included two popular cultivars that are commonly cultivated in Ghana (Aburoheema and Obatanpa GH, coded IM1 and IM6, respectively). The deep knowledge of the genetic diversity and structure of Sahel and coastal West African maize landraces revealed in the present study provides an essential platform for efficient use of these valuable maize gene pools.

## 5. Conclusions

In the present study, we explored the genetic diversity and relationships within and between a maize panel comprising landrace gene pools from Burkina Faso, Ghana, and Togo and compared each to a reference maize population. The analysis of genetic diversity parameters indicated ample genetic diversity in the maize panel. The four multivariate methods were consistent in dividing the maize panel into three distinct genetic groups, each capable of providing different sources of variation for maize genetic enhancement. The genetic divergence of the Burkinabe gene pool was particularly remarkable. It, therefore, clearly represents an invaluable genetic resource that should be exploited to address the overarching goal of improving maize for adaptation to different environments, ecosystems, and stress situations. Overall, the genetic diversity revealed in this study has provided an invaluable resource for future analyses of candidate genes for local adaptations using robust association mapping experiments.

## Figures and Tables

**Table 1:** Diversity statistics based on 5974 DArTseq markers across 208 maize accessions.

Maize Panel	N	H_e_	H_o_	MaF	MAF	PIC
Burkina Faso	58	0.30	0.41	0.77	0.23	0.24
Ghana	43	0.32	0.34	0.77	0.23	0.26
Togo	89	0.36	0.50	0.73	0.27	0.28
Reference population	18	0.36	0.47	0.72	0.28	0.29
Landraces	190	0.37	0.50	0.73	0.27	0.29
Entire Panel	208	0.36	0.50	0.72	0.28	0.29

N/number of accessions; H_e_/expected heterozygosity; H_o_/observed heterozygosity; MaF/major allele frequency; MAF/minor allele frequency; PIC/polymorphic information content.

**Table 2:** Analysis of molecular variance (AMOVA) using 5974 DArTseq markers of the genetic variation among and within four gene pools of 208 maize accessions.

Source	df	SS	MS	Est. Var.	% Var.	*p* Value
Among gene pools	3	54,459.23	18,153.08	168.46	14	0.001
Among individuals	204	420,226.27	2059.93	993.38	80	0.001
Within individuals	208	15,221.5	73.18	73.18	6	0.001
Total	415	489,907.0		1235.02	100	
Fixation index (*F_ST_*)	0.21					0.001
Gene flow (Nm)	1.58					0.001

df/degree of freedom, SS/Sum of square; MS/Mean sum of square, Est. Var./Estimated variance; Var./Variance.

**Table 3:** Measure of genetic population differentiation (*F_ST_*) (lower diagonal), and estimation of gene flow (Nm) (upper diagonal) within and among the four gene pools of maize accessions.

	Burkina Faso	Ghana	Togo	Reference Population
Burkina Faso	-	1.18	1.31	0.98
Ghana	0.27	-	2.63	2.82
Togo	0.24	0.14	-	2.03
Reference population	0.31	0.14	0.17	-
Average *F_ST_*	0.28	0.18	0.18	0.21

## Data Availability

The DArTseq datasets used in the present study have been deposited at the IITA repository. CKAN: http://data.iita.org/dataset/genotypic-data-for-216-maize-inbred-lines-for-diversity-studies.

## Supplementary Materials

The following are available online at https://www.mdpi.com/2073-4425/11/9/1054/s1, Figure S1: Map of West Africa showing the countries of origin of the maize landraces analyzed in this study. Figure S2: Distribution of the 5974 DArTseq GBS makers across the 10 chromosomes of the 208 maize accessions. Table S1: Excel file with the description of the accessions analyzed in this study. Table S2: Excel file showing clustering of the maize 208 accessions based on 5974 DArTseq markers. Table S3: Excel file showing clustering of the four gene pools of maize accessions; Sheet 1. Burkinabe landraces; Sheet 2. Ghanaian landraces; Sheet 3. Togolese landraces; Sheet 4. Reference set. Table S4: List of accessions, and categorization into different clusters by DAPC.
