# Supplementary figures and images for: Genomic Analysis of Selected Maize Landraces from Sahel and Coastal West Africa Reveals Their Variability and Potential for Genetic Enhancement
Source: Genes (Basel). 2020 Sep 7;11(9):1054. doi: 10.3390/genes11091054 (PMC7565678; doi:10.3390/genes11091054)

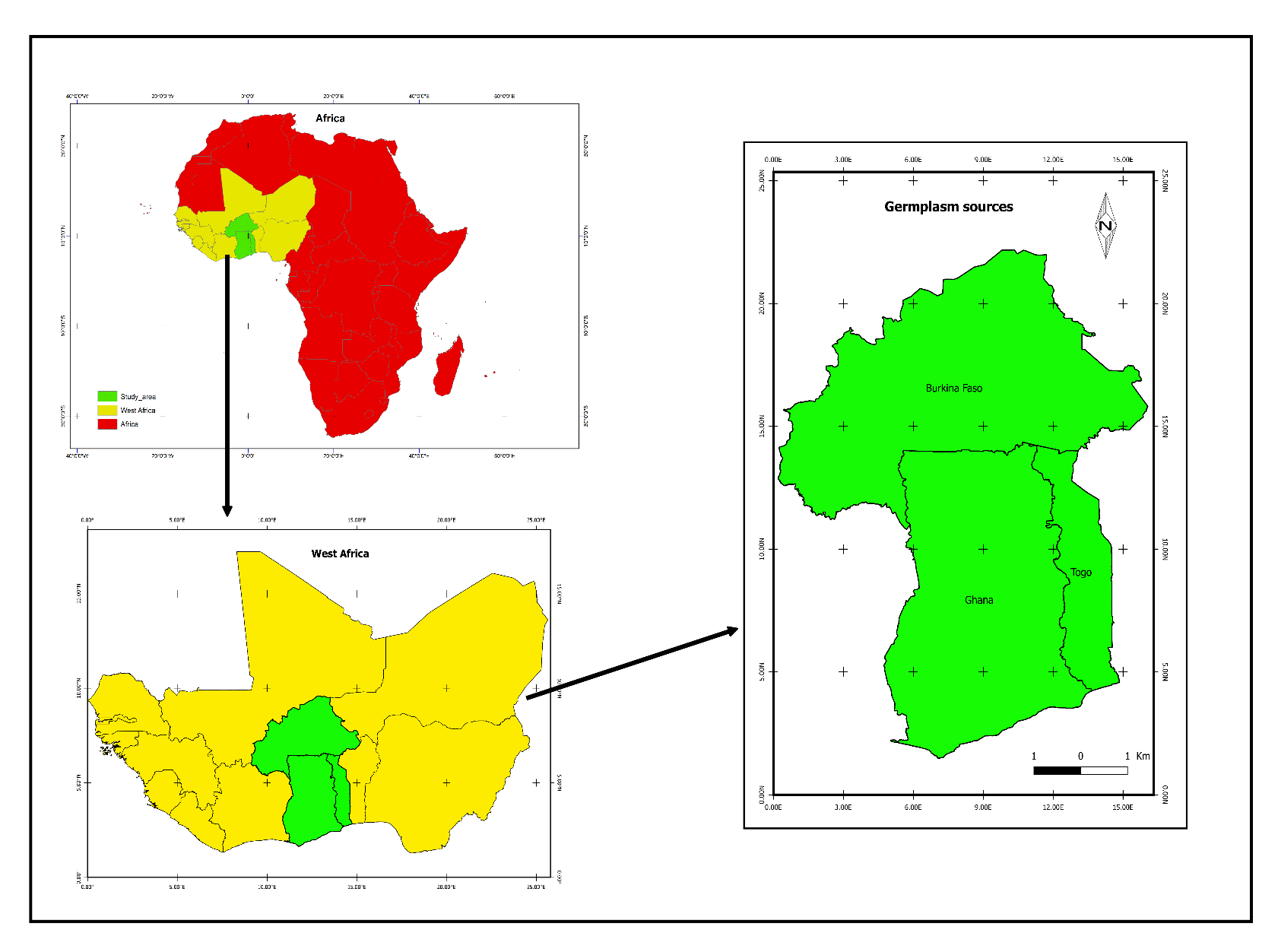

Supplement: Supplementary file 1 [file genes-11-01054-s001.zip › Supplementary Figure S1.png]

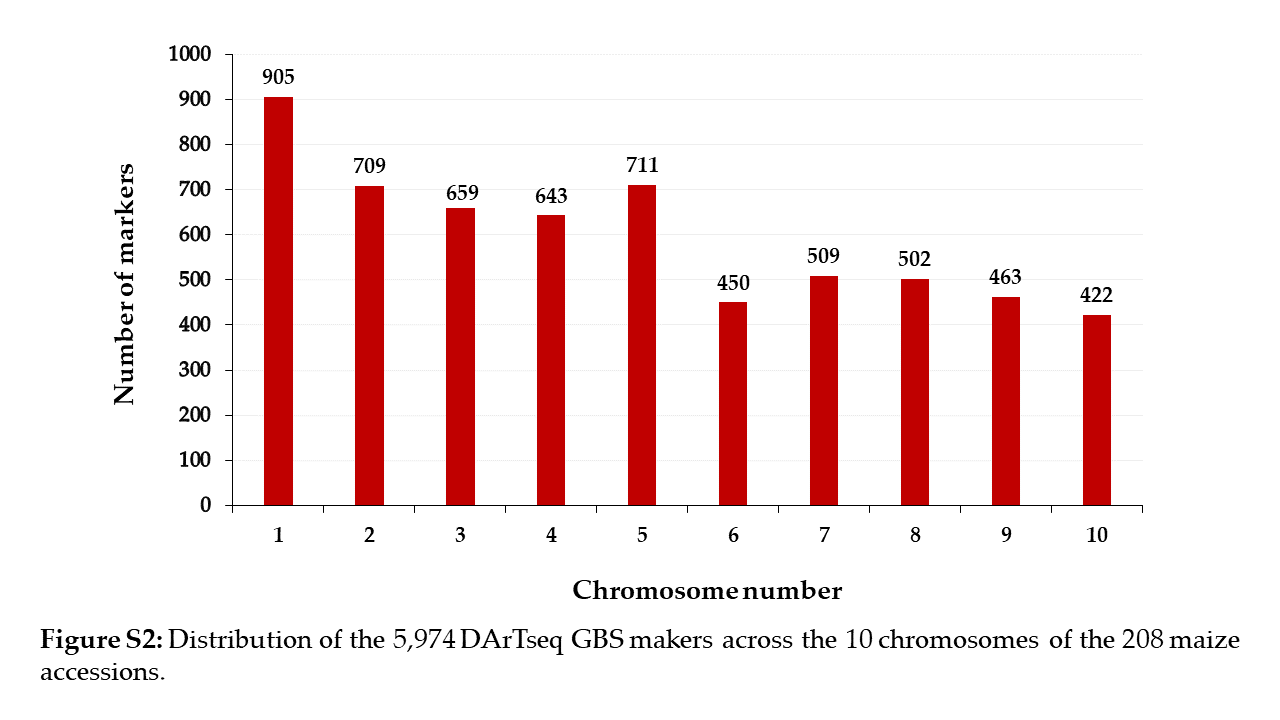

Supplement: Supplementary file 1 [file genes-11-01054-s001.zip › Supplementary Figure S2.png]
